# Inflammatory and chemotactic signals of the brainstem solitary tract mediate the morphine exacerbation of impaired reflex chronotropism in septic rats

**DOI:** 10.3389/fphar.2025.1669284

**Published:** 2026-01-16

**Authors:** Mohamed Abdelnaby, Marwa Y. Sallam, Mai M. Helmy, Hanan M. El-Gowelli, Muddanna S. Rao, Mahmoud M. El-Mas

**Affiliations:** 1 Department of Pharmacology and Toxicology, Faculty of Pharmacy, Alexandria University, Alexandria, Egypt; 2 Department of Anatomy, College of Medicine, Kuwait University, Safat, Kuwait; 3 Department of Pharmacology and Toxicology, College of Medicine, Kuwait University, Safat, Kuwait

**Keywords:** arterial baroreceptors, brainstem, inflammation, morphine, oxidative stress, sepsis

## Abstract

**Introduction:**

The interplay between opioid analgesics and sepsis in intensive care units (ICUs) is multifaceted, often amplifying immune dysregulation and adversely affecting cardiovascular outcomes.

**Aim:**

We investigated whether morphine, the prototypical opioid, influences sepsis-induced impairment of arterial baroreceptor function and the accompanying inflammation.

**Methods:**

Rats were implanted with indwelling catheters in femoral vessels and intracisternal (i.c.) space, and sepsis was induced using the cecal ligation and puncture (CLP) technique. The baroreceptor-mediated control of chronotropic activity was assessed 24 h later in awake rats using the vasoactive method, which relates blood pressure changes caused by i.v. phenylephrine (PE) or sodium nitroprusside (SNP) to respective reciprocal changes in heart rate.

**Results:**

The treatment of sham rats with morphine or induction of sepsis led to comparable attenuations of reflex decrements and increments in chronotropic responses and decreases in slopes of baroreflex curves (baroreflex sensitivity, BRS). The treatment of septic rats with morphine further amplified the decline in reflex bradycardic (BRS_PE_), but not tachycardic (BRS_SNP_), and this exaggerated bradycardia disappeared after (i) systemic blockade of opioid receptors by i.v. morphine or (ii) selective central inhibition of PI3K, MAPK-ERK, MAPK-JNK, NADPHox), or Rho-kinase (ROCK). These pharmacological interventions also attenuated the elevated protein expression of toll-like receptor 4 (TLR4) and monocyte chemoattractant protein-1 (MCP1) in the brainstem nucleus tractus solitarius (NTS) of morphine-treated CLP rats.

**Conclusion:**

Overall, morphine augments the sepsis-induced depression of reflex cardiovagal activity through an opioid receptor sensitive mechanism that engages brainstem inflammatory and chemotactic circuits related to PI3K/MAPK/NADPHox/ROCK signaling.

## 1 Introduction

Sepsis is a life-threatening organ dysfunction that results from dysfunctional host reaction to infection (Singer et al., 2016). Annually, around 45 million patients suffer from sepsis with a 20% death rate (Chiu and Legrand, 2021). Sepsis is one of the most frequent fatal complications encountered in ICUs (Deutschman and Tracey, 2014). Sepsis is associated with dysregulated hemodynamic and cardiovascular functions (Greer, 2015; Sallam et al., 2016). The pathophysiology of sepsis involves activation of toll like receptor-4 (TLR4) (Anderberg et al., 2017) with subsequent release of proinflammatory mediators such as monocyte chemoattractant protein 1 (MCP1) (Zhu et al., 2017; Chen et al., 2023). These mediators cause profound vasodilation, hypotension, and other cardiovascular sequels of septic shock (Nguyen et al., 2006). TLR4 activation causes a consequent upregulation of the MAPK/NF-κB signaling pathway and MCP1 expression, which amplifies the inflammatory response (Chen et al., 2018). The increased expression levels of MCP1 and consequent inflammation disrupt central pathways that are pivotal for cardiovascular autonomic control (Fairchild et al., 2009; Mowry et al., 2021).

The arterial baroreflex pathway serves as a crucial homeostatic mechanism for short-term regulation of blood pressure and other cardiovascular functions (Karim et al., 2023; Salah et al., 2025). Attenuated baroreceptor activity increases the incidence of cardiovascular diseases like hypertension (Gordin et al., 2016) and cardiac arrhythmogenesis and sudden cardiac arrest (Shen and Zipes, 2014). Sepsis disrupts the baroreflex mechanism, resulting in reduced survival time and cardiovascular collapse (Sallam et al., 2021a; Merdji et al., 2022; El-Naggar et al., 2025). The reduction in baroreflex gain in sepsis is likely caused by elevated cytokines and excessive production of nitric oxide and reactive oxygen species (Ulleryd et al., 2017). The nucleus tractus solitarius (NTS) in the lower brainstem serves as the primary medullary site that receives afferent signals from peripheral arterial baroreceptors. The NTS integrates and relays this information to other cardiovascular-regulatory nuclei within the brainstem, as well as to hypothalamic and cortical centers of the central nervous system (Waki et al., 2008; Accorsi-Mendonça and Machado, 2013). Evidence also suggests that afferent vagal neurons are responsive to both pro-inflammatory and anti-inflammatory cytokines, transmitting these immune signals to neurons within the NTS (Amorim et al., 2019). This input triggers autonomic responses that modulate peripheral vascular resistance and elicit rapid adjustments in heart rate (Benarroch, 2008).

Opioids such as morphine and fentanyl are widely used in ICUs for pain control and sedation (Young and De Jong, 2021). The adverse effects of morphine in this context include respiratory depression, peripheral vasodilation, hypotension, and inhibition of arterial baroreceptor reflexes (Shanazari et al., 2011; Krantz et al., 2021; Watso et al., 2022). Although both morphine (Shanazari et al., 2011; Zeinivand et al., 2014) and sepsis (El-Naggar et al., 2025) are each known to impair baroreceptor responsiveness, to our knowledge, no study has examined their combined effects on reflex chronotropic responses to baroreceptor loading and unloading in conscious freely moving rats. Further, this study uniquely explores the role of TLR4 and associated chemotactic and inflammatory pathways within the cardiovascular-regulatory nuclei of the medullary solitary tract in mediating the sepsis-morphine interaction. Hemodynamic, pharmacologic, and protein expression analyses were also integrated to provide novel mechanistic insights into opioid-induced cardiovascular vulnerability during sepsis. The cecal ligation and puncture (CLP) procedure was used in our study to induce sepsis as it closely resembles sepsis in human patients (Gong and Wen, 2019). All the experiments were conducted in fully conscious and freely moving male Wistar rats, pre-instrumented with indwelling femoral and intracisternal catheters.

## 2 Materials and methods

### 2.1 Animals

A total of 60 adult male Wistar rats (200–250 g) were utilized, sourced from the Faculty of Pharmacy animal facility at Alexandria University in Alexandria, Egypt. Rats were kept at an ambient temperature and had a free access to standard rat chow with 19% protein and water. The sample size was estimated *a priori* using power analysis (G*Power 3.1.9.7) (Bate and Clark, 2014) and guided by our previous studies employing similar experimental models (El-Naggar et al., 2025). Before initiating the experiment, the analysis indicated that a minimum of 4 rats per group was required to detect an effect size of 0.90 at an α-error probability of 0.05 and a power (1–β) of 0.80. To ensure adequate statistical robustness, 5-6 rats per group were ultimately included. All experimental procedures and animal handling received approval from the Institutional Animal Care and Use Committee of Alexandria University (Approval No. AU/06.2021.2.6.1.93) and adhered to the ARRIVE guidelines (https://arriveguidelines.org/).

### 2.2 Drugs

The following substances were utilized: Morphine from Masr Pharmaceutical Co. in Cairo, Egypt; Heparin Sodium (5000 IU/mL ampoules) from Nile Pharmaceutical Co., also in Cairo; Naloxone from SERB Pharmaceutical Co. in Paris, France; and Thiopental, branded as Thiopental®, from Biochemie GmbH in Vienna, Austria. Further, wortmannin, PD98056 (PD), and SP600125 (SP) were obtained from Sigma-Aldrich in St. Louis, MO, United States. Diphenyleneiodonium (DPI) and fasudil were provided by Tocris Bioscience in Bristol, UK. PD98056, SP600125, wortmannin, fasudil, and DPI were dissolved in DMSO. Phenylephrine and sodium nitroprusside were also acquired from Sigma-Aldrich in St. Louis, MO, United States.

### 2.3 Sepsis induction by cecal ligation and puncture (CLP)

This procedure was performed as previously described (El-Naggar et al., 2025; Gong and Wen, 2019; El-Naggar et al., 2023). Under thiopental anesthesia (50 mg/kg, i.p.), the rat abdominal region was shaved and disinfected with a betadine. A midline laparotomy of ∼1 cm was made to expose the cecum. The distal one-third of the cecum was ligated, and 3 punctures were made on the same side using a 21-gauge needle. Gentle compression was applied to extrude a small amount of fecal material. The cecum was then returned to the abdominal cavity, and the incision was closed by suturing the abdominal muscle and skin layers.

### 2.4 Intracisternal cannulation (i.c.)

Five days prior to the start of the experiment, i.e., three days before intravascular cannulation and CLP, a stainless steel guide cannula (23 G) was surgically implanted into the cisterna magna of the rats under thiopental anesthesia (50 mg/kg, i.p.), following procedures described in our previous studies (El-Mas et al., 2009; El-Mas et al., 2012). The cannula was carefully advanced between the occipital bone and cerebellum, with its tip positioned in the cisterna magna. It was then secured in place using dental acrylic cement. Successful placement was confirmed by the spontaneous outflow of cerebrospinal fluid. After the surgery, rats were housed individually for recovery.

### 2.5 Intravascular cannulation

The detailed methodology has been previously described (El-Mas et al., 2009; El-Mas et al., 2012). Following CLP or sham surgery, vascular catheters were implanted using a catheter consisting of a 5 cm segment of polyethylene-10 tubing fused to a 15 cm segment of polyethylene-50 tubing. These catheters were inserted into the inferior vena cava and abdominal aorta via the femoral vein and artery for intravenous drug administration and arterial blood pressure monitoring, respectively. The intravascular portion was composed of the polyethylene-10 segment. The catheters were then tunneled subcutaneously and exteriorized at the back of the neck between the scapulae. They were then flushed with heparinized saline (0.2 mL; 100 U/ml) and secured using stainless steel pins. On the following day, the arterial catheter was connected to a pressure transducer (model P23XL; Astro-Med, West Warwick, RI, United States), linked via an MLAC11 Grass adapter cable to a computerized data acquisition system (LabChart-7 Pro software, PowerLab 4/35, model ML866/P; AD Instruments Pty Ltd., Castle Hill, Australia) for continuous recording and analysis of blood pressure and heart rate. It is noteworthy that thiopental, the anesthetic drug used in this study, is an ultra-short-acting barbiturate that permits rapid recovery, typically within 30–40 min after i.p. administration. Normal spontaneous behavior and body reflexes are generally restored within 2 h after anesthesia (Tanaka et al., 2008; Kushikata et al., 2011). In our study, baroreflex assessment was conducted 24 h after intravascular cannulation, when the rats were fully awake, freely moving in their home cages, and had resumed normal eating and drinking habits. No differences were observed between the CLP and sham-operated groups in their recovery from anesthesia.

### 2.6 Measurement of baroreflex gain (Oxford method)

Baroreflex sensitivity (BRS) was assessed at the end of the experiment using the vasoactive (Oxford) method, as previously described (El-Mas and Abdel-Rahman, 1993; El-Mas et al., 2011; El-Naggar et al., 2024; El-Naggar et al., 2025). This approach evaluates reflex bradycardic and tachycardic responses to peripheral blood pressure changes induced by randomized intravenous bolus injections of PE or SNP (1–16 μg/kg, administered every 5 min), respectively. Baroreflex response curves were generated by measuring mean arterial pressure (MAP) against heart rate (HR) values before and after drug administration. Peak changes in MAP (ΔMAP) and HR (ΔHR) were determined, and the slope of the resulting linear regression curve was used to quantify BRS.

### 2.7 Immunohistochemistry

This was used to measure the expression of TLR4 and MCP1 in the brain medullary neurons of the nucleus tractus solitarius (NTS) (Sallam et al., 2019; Sallam et al., 2021a). Tissues were fixed in 10% formalin solution for 1 day, dehydrated in a graded series of ethanol (70, 95% and 100%), then embedded in paraffin. Tissue sections of 5 µm thickness of the rostral NTS (−12.3 mm relative to bregma) (Paxinos and Watson, 1986; Abuiessa et al., 2020) were placed on positively charged adhesion microscope slides, deparaffinized in xylene and rehydrated in a graded series of ethanol (100, 95% and 70%). Slides were then rinsed gently with PBS and drained. Heat-induced epitope retrieval was done by immersing slides in Coplin jars containing 10 mM citrate buffer solution and incubation in a microwave at power100 for 1min then power 30 for 9 min. The sections were subsequently rinsed with 1× TBST (50 mM Tris/HCl, pH 7.4, 150 mM NaCl, 0.1% Tween 20). To block endogenous peroxidases, a 3% hydrogen peroxide solution was applied, followed by washing with 1× TBST. Following that, a universal protein blocking solution was applied for 20 min. The appropriate primary monoclonal antibodies (rabbit anti-TLR4 & rabbit anti-MCP1) were diluted (1:300) as instructed by the manufacturer and applied to the slides then slides were incubated over night at 4 °C. Following this incubation, the slides were washed with 1× TBST, rinsed, and subsequently incubated for 30 min with the secondary antibody (HRP conjugate). The chromogen 3,3′-diaminobenzidine was prepared and applied in accordance with the manufacturer’s instructions for protein visualization. Each slide was counterstained with haematoxylin and dipped in ascending concentrations of alcohol and then xylene. OptikamB9 digital camera was used to take images (Optika® microscopes, Italy) followed by quantification of the immunohistochemical signals of expressed proteins in the NTS area of the brainstem using Fiji ImageJ software version 1.51n (National Institutes of Health, Bethesda, Maryland, United States). The quantification procedure involved measuring the stain intensity in 6–9 distinct rNTS sections from each hemisphere. The mean intensity values from both hemispheres were then averaged to obtain a representative value for each animal, ensuring consistency and minimizing regional variability.

### 2.8 Protocols and experimental groups

**FIGURE 1:**
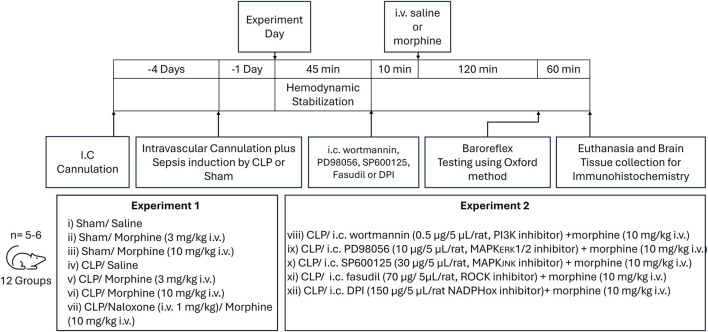
Diagrammatic representation of the timeline of surgical procedures, drug regimens and experimental groups.

This study investigated the dose-dependent effects of systemic morphine administration on baroreflex activity in septic and sham operated rats. Male Wistar rats were randomly divided into seven experimental groups (n = 5-6 per group): (i) sham/saline, (ii) sham/morphine (3 mg/kg), (iii) sham/morphine (10 mg/kg), (iv) CLP/saline, (v) CLP/morphine (3 mg/kg), (vi) CLP/morphine (10 mg/kg), and (vii) CLP/naloxone (1 mg/mL)/morphine (10 mg/kg). Following a stabilization period of at least 45 min, either saline or morphine was administered intravenously. Two hours later, baroreflex response curves to PE and SNP were generated as previously described. These treatments were randomly assigned to the respective rat groups using the simple randomization sequence, which is based on a single sequence of random assignments. Rats were excluded from the experiment if any of the arterial or venous catheters were blocked or disconnected. At the end of the experiment, animals were euthanized with an overdose of thiopental (100 mg/kg). The brainstem was then collected, fixed in 10% formaldehyde, and processed for immunohistochemical analysis of MCP1 and TLR4 protein expression. A schematic diagram of the experimental timeline, including surgical procedures and drug administration protocols, is shown in Figure 1. Blinding of the experimental procedures was not feasible, as the same researcher conducted both the surgical interventions and drug administrations.

To further examine the involvement of inflammatory PI3K/MAPK/NADPHox/ROCK pathway in the interaction between sepsis and morphine, five additional CLP rat groups (n = 5-6 per group) were included randomly assigned to receive one of the following i.c. regimens followed by morphine (10 mg/kg, i.v.): (i) PD98056 (10 μg/5 μL/rat, a MAPK/ERK1/2 inhibitor), (ii) SP600125 (30 μg/5 μL/rat, a MAPK/JNK inhibitor), (iii) wortmannin (0.5 μg/5 μL/rat, a PI3K inhibitor), (iv) fasudil (70 μg/5 μL/rat, a ROCK inhibitor), and (v) diphenyleneiodonium (DPI; 150 μg/5 μL/rat, a NADPHox inhibitor). The dosing regimens were selected based on previously published studies (Fujita et al., 2007; Sallam et al., 2016; Kumar and Bansal, 2018). A 10-min interval was maintained between the administration of the inhibitor and morphine in each treatment protocol. Following drug administration, baroreflex response curves to PE and SNP were constructed as previously described. At the end of hemodynamic monitoring, rats were euthanized via thiopental overdose (100 mg/kg), and brainstems were collected, fixed in 10% formaldehyde, and processed for immunohistochemical analysis of MCP1 and TLR4 protein expression, as detailed earlier.

### 2.9 Statistical analysis

Data are presented as means ± standard error of the mean (S.E.M.). BRS was evaluated by assessing the relationship between changes in MAP and corresponding changes in HR using linear regression analysis for each animal, as previously described (Paxinos and Watson, 1986; El-Mas and Abdel-Rahman, 1992; El-Mas et al., 2001; Abuiessa et al., 2020; Sallam et al., 2021a). The slope of the regression line, expressed in beats per minute per mm Hg, was used as an index of BRS. Statistical comparisons among groups were performed using one-way or repeated-measures ANOVA, followed by Tukey’s *post hoc* test for multiple comparisons. The analysis was performed using GraphPad Prism software, version. 10.6.1. Probability levels less than 0.05 were considered significant.

## 3 Results

### 3.1 Morphine dose dependently aggravates CLP- induced baroreflex dysfunction

**FIGURE 2:**
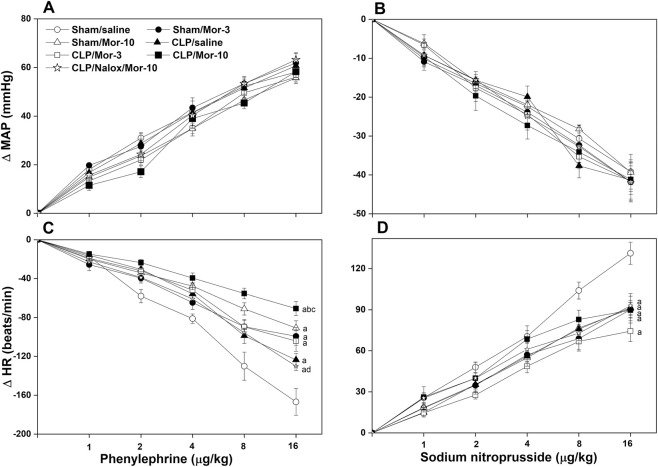
Effect of i.v. morphine (3 or 10 mg/kg) on increases and decrease in MAP elicited by phenylephrine (panel **(A)**) and sodium nitroprusside (panel **(B)**) (1–16 μg/kg each), respectively (upper panels) and reciprocal changes in HR (panels **(C, D)**, respectively) in conscious sham operated rats and CLP rats. The effect of opioid receptor antagonism by naloxone (1 mg/kg i.v.) on morphine responses in CLP rats is also shown. Values are means ± SEM of five to six observations. ^a^P < 0.05 vs. “sham/saline”, ^b^P < 0.05 vs. “CLP/saline”, ^c^P < 0.05 vs. “sham/morphine-10”, ^d^P < 0.05 vs. “CLP/Morphine-10”.

**FIGURE 3:**
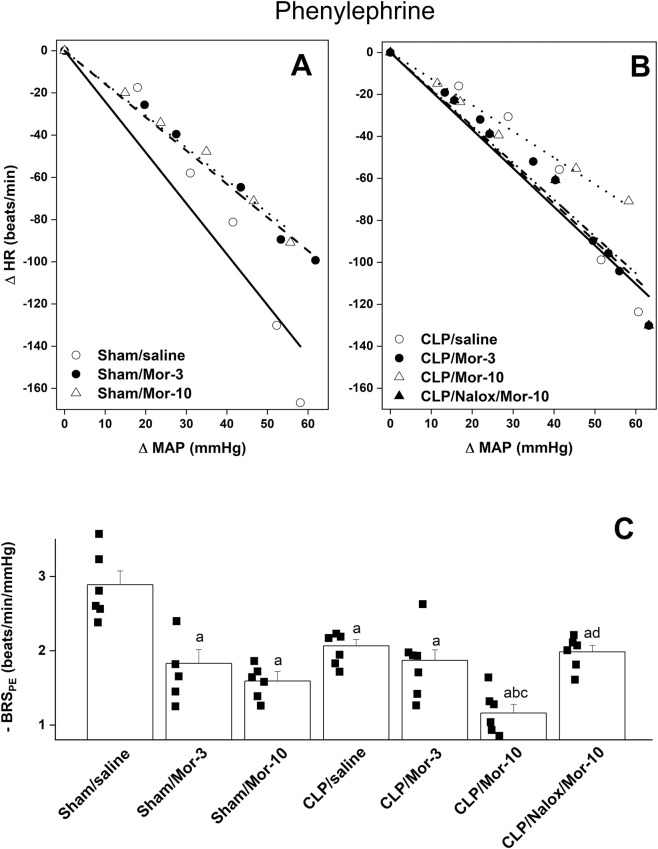
Effect of i.v. morphine (3 or 10 mg/kg) on baroreflex curves generated by phenylephrine (1–16 μg/kg) and baroreflex sensitivity (BRSPE, slopes of the curves) in conscious sham operated rats and CLP rats. The effect of opioid receptor antagonism by naloxone (1 mg/kg i.v.) on morphine responses in CLP rats is also shown. In **(A)**, the regression lines represent the following groups: sham/saline (solid line), sham/morphine-3 (dashed line), sham/morphine-10 (dotted line). In **(B)**, the regression lines represent the following groups: CLP/saline (solid line), CLP/morphine-3 (dashed line), CLP/morphine-10 (dotted line), and CLP/naloxone/morphine-10 (dash-dotted line). In **(C)**, BRSPE. Values are means ± SEM of five to six observations. The repeated measures ANOVA followed by the Tukey’s post hoc test were employed to test for statistical significance. ^a^P < 0.05 vs. “sham/saline”, ^b^P < 0.05 vs. “CLP/saline”, ^c^P < 0.05 vs. “sham/morphine-10”, ^d^P < 0.05 vs. “CLP/Morphine-10”.

**FIGURE 4:**
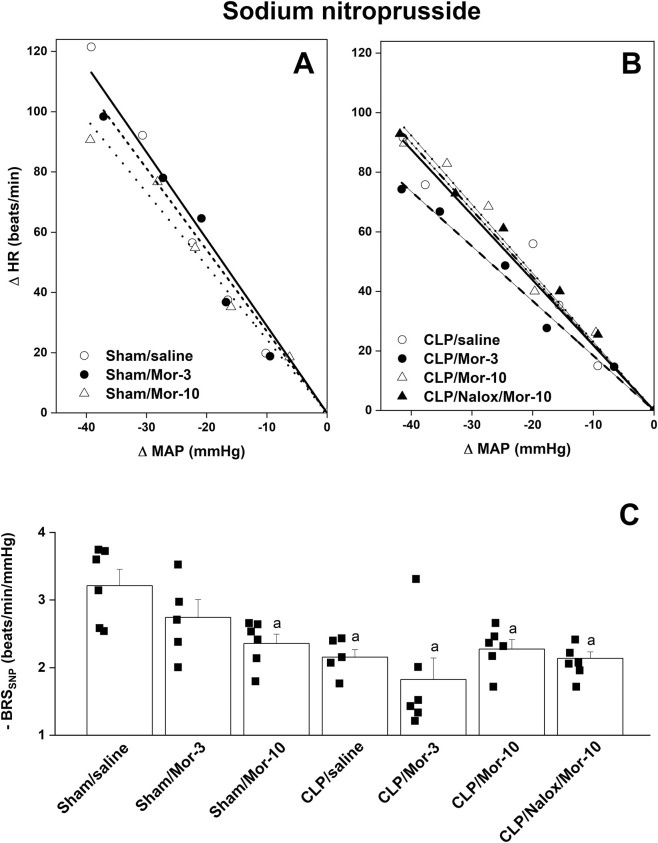
Effect of i.v. morphine (3 or 10 mg/kg) on baroreflex curves generated by sodium nitroprusside (1–16 μg/kg) and baroreflex sensitivity (BRSSNP, slopes of the curves) in conscious sham operated rats and CLP rats. The effect of opioid receptor antagonism by naloxone (1 mg/kg i.v.) on morphine responses in CLP rats is also shown. In **(A)**, the regression lines represent the following groups: sham/saline (solid line), sham/morphine-3 (dashed line), sham/morphine-10 (dotted line). In **(B)**, the regression lines represent the following groups: CLP/saline (solid line), CLP/morphine-3 (dashed line), CLP/morphine-10 (dotted line), and CLP/naloxone/morphine-10 (dash-dotted line). In **(C)**, BRSSNP. Values are means ± SEM of five to six observations. The repeated measures ANOVA followed by the Tukey’s post hoc test were employed to test for statistical significance. ^a^P < 0.05 vs. “sham/saline”.

Table 1 shows that prior to saline or morphine administration, all CLP groups demonstrated significant decreases and increases in baseline MAP and HR values compared to respective sham values. Figures 2–4 illustrate the effect of morphine on baroreflex curves constructed by the vasoactive method in sham and CLP rats. In all rat groups, intravenous administration of graded doses of PE and SNP (1–16 μg/kg each) elicited dose-dependent increases and decreases in MAP (Figures 2A,B, respectively, accompanied by corresponding reciprocal changes in HR (Figures 2C,D). Compared with sham rats, the induction of sepsis by CLP did not affect MAP responses to PE or SNP but significantly blunted the corresponding reflex decreases and increases in HR, respectively (Figure 2), indicating impaired baroreflex-mediated heart rate regulation in septic rats. Comparisons of the slopes of the regression lines, which represent BRS, showed that the induction of sepsis by CLP caused significant decreases in baroreflex sensitivity measured by PE (BRS_PE_, −2.06 ± 0.08 vs. −2.88 ± 0.18 beats/min/mmHg, 95% CI of diff. 0.193 to 1.455, *p =* 0.004, Figure 3C) and SNP (BRS_SNP_, −2.15 ± 0.11 vs. −3.21 ± 0.24 beats/min/mmHg, 95% CI of diff. 0.1252 to 1.986, *p =* 0.018, Figure 4C) compared with control (sham-operated) values.

**TABLE 1:** Baseline measurements of mean arterial pressure (MAP, mmHg) and heart rate (HR, beats/min) recorded in conscious sham or CLP rats before the administration of any saline or drug treatments.

Group	MAP	HR
Sham/Saline	106 ± 4	388 ± 13
Sham/Mor-3	104 ± 3	369 ± 21
Sham/Mor-10	105 ± 4	377 ± 12
CLP/Saline	93 ± 4*	419 ± 15*
CLP/Mor-3	92 ± 5*	423 ± 16*
CLP/Mor-10	91 ± 3*	427 ± 11*
CLP/Nalox/Mor-10	93 ± 4*	444 ± 10*

Values are means ± SEM, of five to six observations. *P < 0.05 vs. respective sham values.

Figure 3 demonstrates that the administration of morphine at 10 mg/kg to CLP rats further worsened the sepsis-associated impairment in reflex bradycardia, leading to an additional reduction in BRS_PE_ compared with CLP alone (95% CI of diff. 0.2731 to 1.536, *p* = 0.001, Figure 3C). The amplifying effect of morphine appears to be specific to the reflex bradycardic response, as the same dose did not result in any further decrease in BRS_SNP_ in septic rats (95% CI of diff. −1.047 to 0.8132, *p* = 0.999, Figure 4C). Notably, the morphine effects were evaluated relative to the corresponding post-saline measurements, as baroreflex activity was not assessed before administration of morphine or saline. The depressant effect of morphine on BRS_PE_ in CLP rats was abolished by prior co-administration of the opioid receptor antagonist naloxone (Figure 3).

**FIGURE 5:**
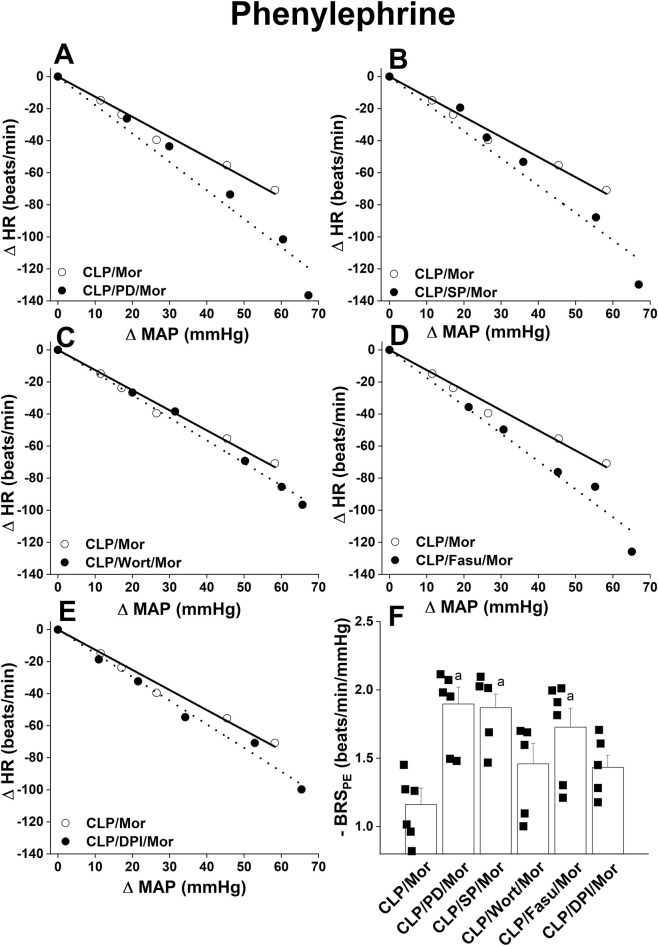
Effect of i.c. pretreatment with PD98056 (10 μg/5 μL/rat, MAPKERK1/2 inhibitor, panel **(A)**), SP600125 (30 μg/5 μL/rat, MAPKJNK inhibitor, panel **(B)**), wortmannin (0.5 μg/5 μL/rat, PI3K inhibitor, panel **(C)**), fasudil (70 μg/5μL/rat, ROCK inhibitor, panel **(D)**) or DPI (150 μg/5 μL/rat NADPHox inhibitor, panel **(E)**) on baroreflex curves generated by phenylephrine (1–16 μg/kg) and baroreflex sensitivity (BRS curves) in conscious CLP rats treated with morphine (10 mg/kg). In **(F)**, BRSPE. Values are means ± SEM of five to six observations. The repeated measures ANOVA followed by the Tukey’s post hoc test were employed to test for statistical significance. ^a^P < 0.05 vs. “CLP/morphine”.

**FIGURE 6:**
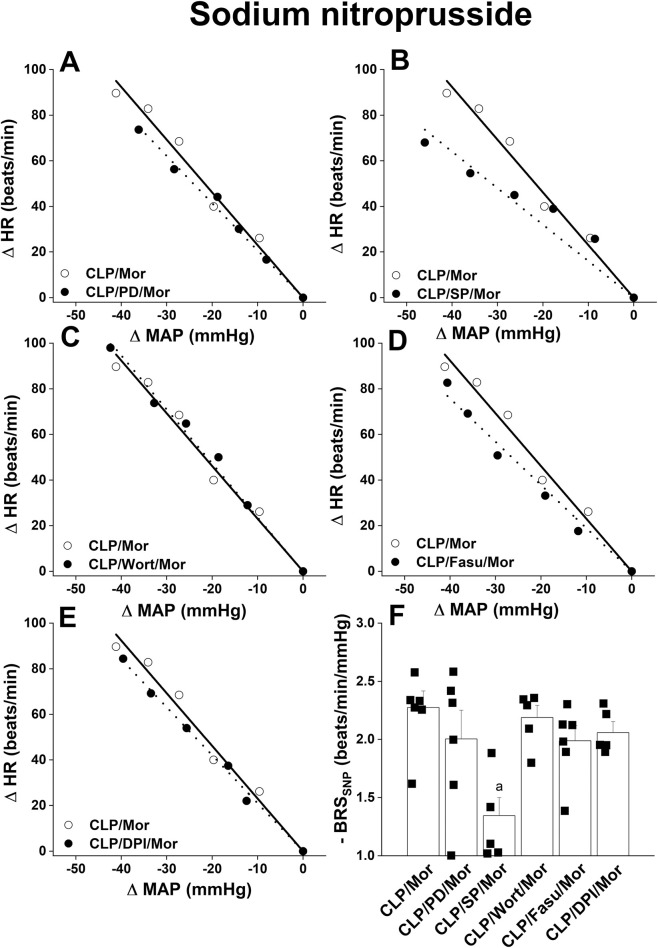
Effect of i.c. pretreatment with PD98056 (10 μg/5 μL/rat, MAPKERK1/2 inhibitor, panel **(A)**), SP600125 (30 μg/5 μL/rat, MAPKJNK inhibitor, panel **(B)**), wortmannin (0.5 μg/5 μL/rat, PI3K inhibitor, panel **(C)**), fasudil (70 μg/5μL/rat, ROCK inhibitor, panel **(D)**)or DPI (150 μg/5 μL/rat NADPHox inhibitor, panel **(E)**) on baroreflex curves generated by sodium nitroprusside (1–16 μg/kg) and baroreflex sensitivity (BRS curves) in conscious CLP rats treated with morphine-10. In **(F)**, BRSSNP. Values are means ± SEM of five to six observations. The repeated measures ANOVA followed by the Tukey’s post hoc test were employed to test for statistical significance. ^a^P < 0.05 vs. “CLP/morphine”.

Pharmacological antagonist studies were undertaken to investigate the role of central inflammatory and oxidative pathways in the morphine-BRS_PE_ interaction in septic rats. Figure 5 shows that prior i.c. administration of the pharmacologic inhibitor of MAPK-ERK (PD98056, 10 μg/rat, Figure 5A), MAPK-JNK (SP600125, 30 μg/rat, Figure 5B), or ROCK (fasudil, 70 μg/rat, Figure 5D) caused downward shifts in the baroreflex curves generated by PE in morphine-treated CLP rats and significant increases in BRS_PE_ (Figure 5F). No such effect was demonstrated after central inhibition of PI3K (wortmannin, 0.5 μg/rat, Figures 5C–F) or NADPHox (DPI, 150 μg/rat, Figures 5E,F). Further, none of these inhibitors affected BRS_SNP_ in CLP-morphine-treated rats, except for a significant reduction caused by the MAPK-JNK inhibitor SP600125 (Figure 6).

### 3.2 Modulation of sepsis-morphine baroreflex interaction by central inflammation and chemotaxis

**FIGURE 7:**
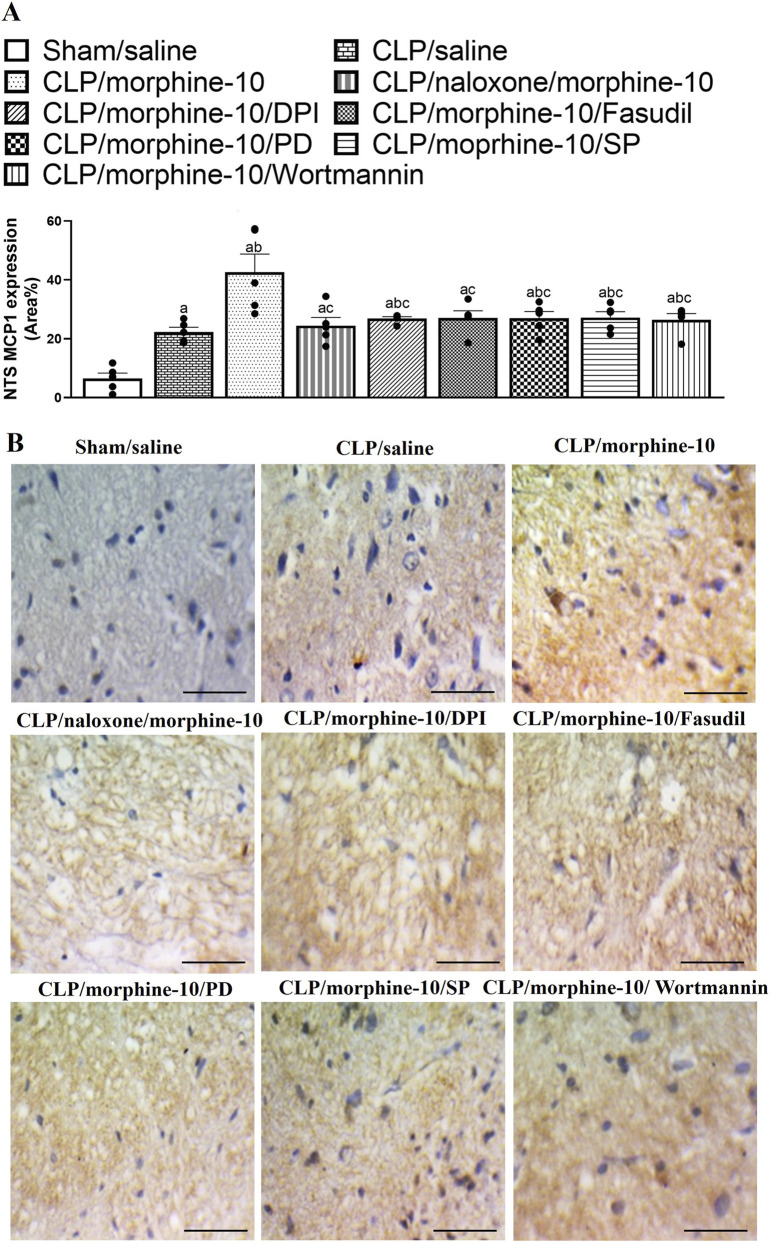
**(A)** illustrates the effects of naloxone (1 mg/kg i.v.) or i.c. administration of wortmannin (0.5 μg/5 μL/rat, PI3K inhibitor), PD98056 (10 μg/5 μL/rat, MAPK_ERK1/2_ inhibitor), SP600125 (30 μg/5 μL/rat, MAPK_JNK_ inhibitor), DPI (150 μg/5 μL/rat NADPHox inhibitor) or fasudil (70 μg/5μL/rat, ROCK inhibitor) on morphine (10 mg/kg)-evoked rises in immunohistochemical protein expressions of MCP1 in the nucleus tractus solitarius (NTS) of septic rats. Representative images of the NTS expression of MCP1 are shown in **(B)**. Values are means ± SEM of five observations. ^a^P < 0.05 vs. “sham/saline”, ^b^P < 0.05 vs. “CLP/saline”, ^c^P < 0.05 vs. “CLP/morphine-10”.

**FIGURE 8:**
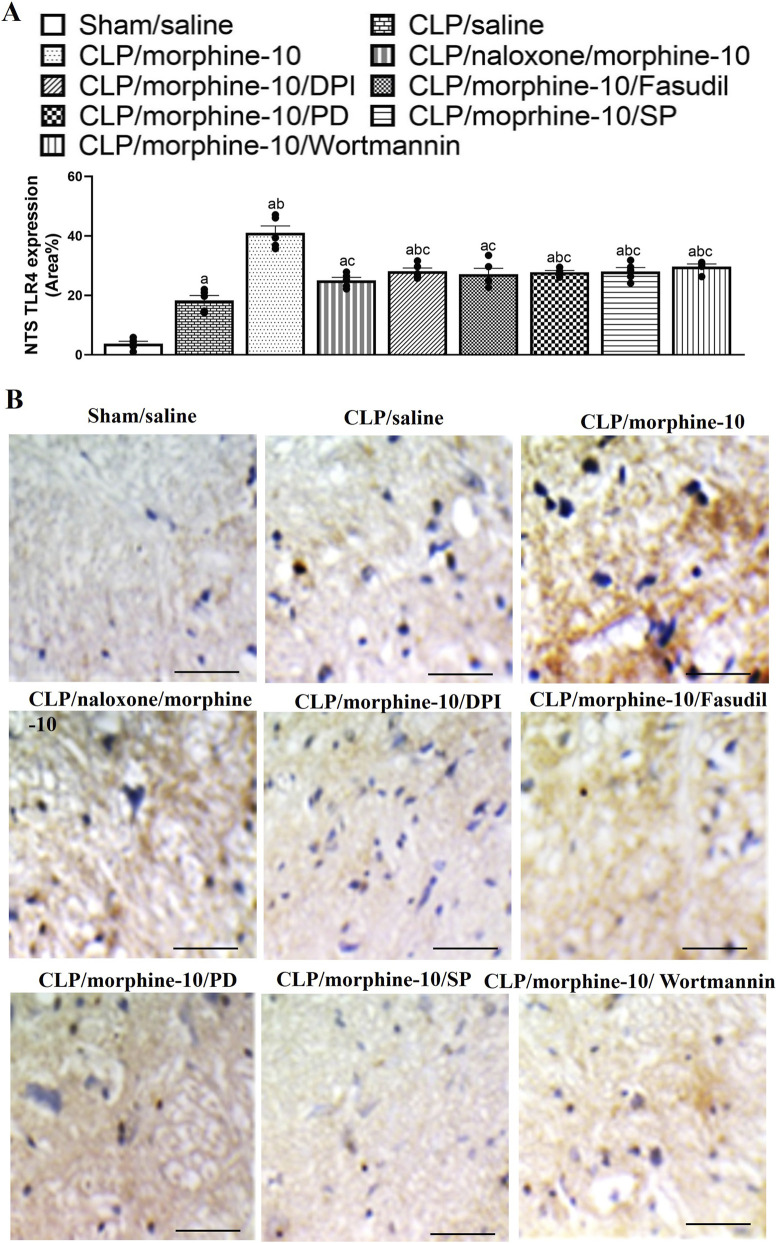
**(A)** illustrates the effects of naloxone (1 mg/kg i.v.) or i.c. administration of wortmannin (0.5 μg/5 μL/rat, PI3K inhibitor), PD98056 (10 μg/5 μL/rat, MAPK_ERK1/2_ inhibitor), SP600125 (30 μg/5 μL/rat, MAPK_JNK_ inhibitor), DPI (150 μg/5 μL/rat NADPHox inhibitor) or fasudil (70 μg/5μL/rat, ROCK inhibitor) on morphine (10 mg/kg)-evoked rises in immunohistochemical protein expressions of TLR4 in the nucleus tractus solitarius (NTS) of septic rats. Representative images of the NTS expression of TLR4 are shown in **(B)**. Values are means ± SEM of five observations. ^a^P < 0.05 vs. “sham/saline”, ^b^P < 0.05 vs. “CLP/saline”, ^c^P < 0.05 vs. “CLP/morphine-10”.

**FIGURE 9:**
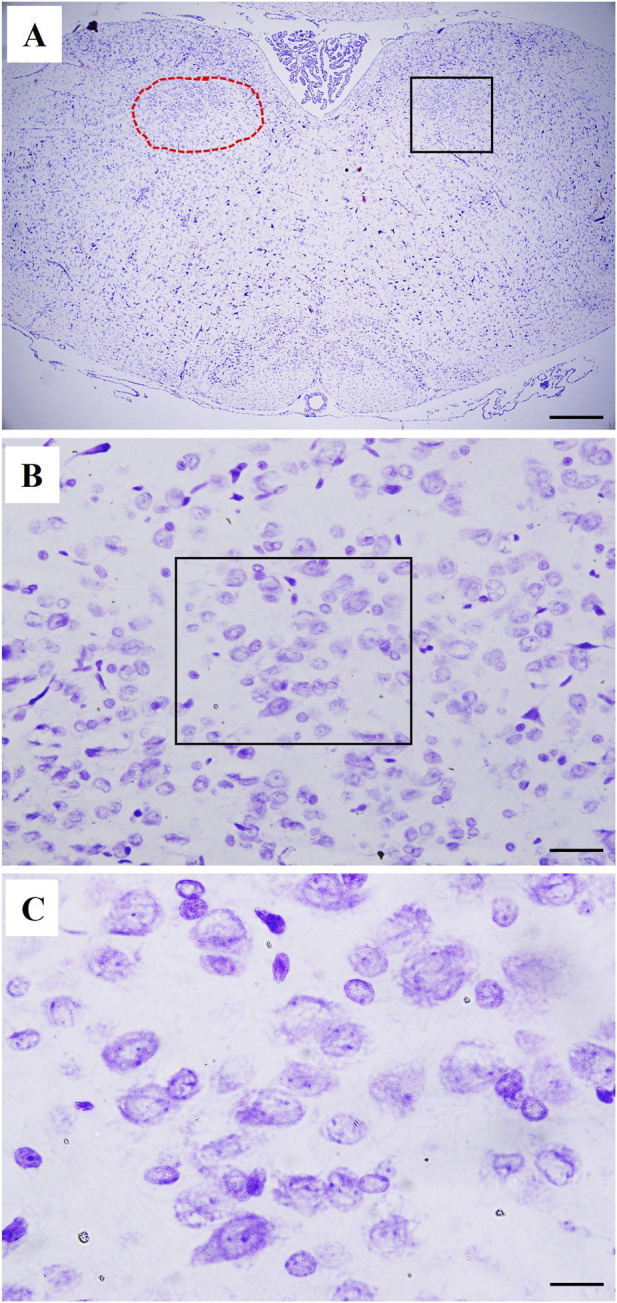
**(A)** is a photomicrograph of a coronal section of the medulla oblongata corresponding to Plate no 136 (−12.36 mm from bregma) in the rat brain atlas of Paxinos and Watson (Paxinos and Watson, 1986; Nakano et al., 2015; Abuiessa et al., 2020; Sallam et al., 2021b), stained with cresyl violet. The nucleus tractus solitarius (NTS) is outlined on the left side with a dotted red line. On the right side, the area enclosed within the square box represents the region selected for further analysis, which is illustrated in Figures 8 and 9 and shown at a higher magnification in **(B)** (scale bar represents 500 µm). **(B)** shows a magnified view of the NTS, highlighting the morphology of its neurons as revealed by cresyl violet staining (scale bar corresponds to 40 µm). **(C)** depicts an even higher magnification of the area enclosed by the square box in panel B, allowing a more detailed visualization of the neuronal features (scale bar represents 20 µm).

The effect of morphine on the immunohistochemical expression of inflammatory (TLR4) and chemotactic (MCP1) signals in the NTS neuronal pools of septic rats are shown in Figures 7, 8. The induction of sepsis resulted in significant increases in the NTS expression of MCP1 (Figure 7) and TLR4 (Figure 8) and these effects were further augmented in NTS tissues of morphine-treated CLP rats. The enhancing effect of morphine on MCP1/TLR4 expression was abolished following the systemic administration of naloxone, suggesting the importance of µ-opioid receptors in mediating the morphine response. Moreover, the chemotactic and inflammatory actions of morphine in the solitary tract were ameliorated after i.c. administration of PD98056, SP600125, wortmannin, fasudil or DPI (Figures 7, 8). Representative images of the NTS expression of MCP1 and TLR4 are presented in Figures 7, 8, respectively. Figure 9 shows a cresyl violet–stained coronal section of the medulla oblongata (Plate 136, −12.36 mm from bregma) (Paxinos and Watson, 1986; Nakano et al., 2015; Abuiessa et al., 2020; Sallam et al., 2021b).

## 4 Discussion

This study reports on the interaction between morphine and sepsis induced by CLP on arterial baroreceptor control in awake rats and possible underlying molecular mechanisms. The data demonstrates that both morphine treatment and sepsis independently reduce baroreceptor-mediated control of heart rate, and that morphine further amplifies the sepsis-induced impairment of cardiovagal reflex control. Immunohistochemical studies reveal that this effect of morphine is mediated via the upregulation of central TLR4 and MCP1 expressions. Pharmacological inhibition of opioid receptors or key signaling inflammatory (MAPK-ERK or MAPK-JNK) or oxidative molecules (Rho-kinase) reversed both the exaggerated reflex bradycardic impairment and upregulated inflammatory signals. These findings highlight that morphine worsens sepsis-induced baroreflex dysfunction via opioid receptor-dependent activation of brainstem inflammatory and chemotactic signaling pathways.

Physiologically, arterial baroreceptors function as a key homeostatic mechanism that buffers abrupt changes in BP chiefly through modulating central sympathetic and vagal outflows to cardiovascular structures (El-Mas et al., 2001; Stocker et al., 2019). Baroreceptor dysfunction is commonly observed in cardiovascular diseases such as hypertension and myocardial infarction and is frequently correlated with poor prognosis (Gerritsen et al., 2001; Shen et al., 2004; Shi et al., 2007). The impairment of baroreflex activity is also provoked by the septic challenge in both clinical (Sharshar et al., 2003) and experimental studies (Milanez et al., 2022). The diminished baroreflex activity has been blamed for hemodynamic instability and increased incidence of morbidity and mortality in septic shock (Carrara et al., 2020; Merdji et al., 2022). A restraining influence of arterial baroreceptors against septic sequels is also supported by the observation that the septic hypotension and cardiovascular collapse are heightened after surgical interruption of arterial baroreceptor afferents (Amorim et al., 2020). Consistent with these earlier reports, the assessment of baroreceptor function in the present study using the vasoactive method revealed clear evidence of blunted reflex bradycardic and tachycardic responses compared to sham-operated controls.

The present study builds upon our earlier work (Abdelnaby et al., 2025) by extending the investigation of opioid–sepsis interactions to a distinct and more refined experimental framework. Unlike the previous study (Abdelnaby et al., 2025), which focused on systemic cardiovascular parameters like blood pressure, cardiac contractility, and heart rate variability, the current work specifically aims to examine the influence of morphine on arterial baroreflex dysfunction induced by sepsis. By combining direct baroreflex sensitivity assessment in awake animals with intracisternal pharmacological manipulation and molecular analyses within the NTS, this study provides novel, site-specific mechanistic insight into central pathways linking opioid exposure to reflex cardiovascular regulation during sepsis. Addressing this question is especially important as opioids are commonly administered in ICUs to control pain and mitigate stress in critically ill patients, including those suffering from sepsis (Molina, 2006; Wang et al., 2025). Conflicting findings have been reported concerning the effect of opioids on baroreflexes, with studies reporting both inhibitory (Maley and Elde, 1982; Wang and Li, 1988; Zeinivand et al., 2014) and stimulatory effects (Shanazari et al., 2011). These discrepancies are thought to arise from variations in the subject under investigation and dose and duration of opioids. Our finding that morphine inhibited baroreflex-mediated bradycardia, but not tachycardia, in septic rats likely reflects a differential influence of morphine on vagal versus sympathetic arms of the autonomic reflex. This premise is based on reports that baroreflex-induced bradycardia is predominantly mediated by activation of cardiac vagal efferents, whereas reflex tachycardia largely depends on sympathetic excitation (El-Mas et al., 2001; El-Mas et al., 2002; Moore et al., 2022). The presumed selective interaction of morphine with central vagal pools receives support from the observations that μ-opioid receptors are expressed in brainstem vagal nuclei, e.g., nucleus ambiguous and dorsal motor nucleus of the vagus, and that morphine reduces excitatory input to these cardiac vagal neurons (Mansour et al., 1995; Nomura et al., 1996; Venkatesan et al., 2003). Moreover, the present finding that the BRS_PE_ depressant effect of morphine in septic rats was attenuated in rats pretreated with the μ-opioid receptor antagonist naloxone reinforces the critical involvement of μ-opioid receptors in mediating the morphine-induced reduction of vagally driven baroreflex responses.

Pharmacological interventions were employed to explore the roles of central PI3K/MAPKs and NADPHox/ROCK pathways in the amplifying effect of morphine on sepsis-induced impairment of BRS_PE_. PI3K and MAPK play pivotal roles in the pathophysiology of the inflammatory response to sepsis and associated cardiovascular, autonomic and baroreceptor dysfunctions (El-Naggar et al., 2024; El-Naggar et al., 2025). NADPHox and ROCK are oxidative signals that cross talk with the inflammatory pathway to enhance microglial activation, impair cardiovascular functions and disrupt autonomic reflexes (Liu et al., 2025). In the present study, the inhibitors of these molecular targets were administered into the cisterna magna, a cerebellomedullary area through which cerebrospinal fluid bathes key autonomic nuclei of the brainstem including (Miura and Reis, 1969; Paxinos and Watson, 1986; Chalmers and Pilowsky, 1991; Misu et al., 1996; Abuiessa et al., 2020). Our findings demonstrated that the central inhibition of MAPK-ERK, MAPK-JNK, or ROCK by i.c. PD98056, SP600125 and fasudil, respectively, abolished the depressant effect of morphine on baroreceptor-mediated bradycardia in CLP rats, inferring the involvement of these central pathways in the morphine-BRS_PE_ interaction. By contrast, PI3K or Rho-kinase appear to serve no role in this setting because the inhibition of these molecules by wortmannin and DPI, respectively, failed to reverse the BRS_PE_ depressant effect of morphine. Notably, although MAPKs are frequently activated downstream of PI3K signaling, they can also be triggered via PI3K-independent pathways (Wang et al., 2025). Evidence also exists that ROCK can be activated through mechanisms independent of Rho GTPases (Croft and Olson, 2006).

TLR4 and MCP1 are pivotal mediators of the innate immune response and sepsis pathogenesis. TLR4 causes downstream activation of MAPKs through MyD88-and TRIF-dependent pathways, that leads to the production of cytokine storm triggered by sepsis (Wang et al., 2025). In parallel, MCP1 is a chemokine that recruits monocytes, memory T cells, and dendritic cells to sites of infection and inflammation, thereby amplifying the immune response (Chen et al., 2023). TLR4 activation during sepsis acts through the MAPK/NF-κB pathway to upregulate MCP1 production, which serves as a positive feedback loop that promotes inflammation (Chen et al., 2018). Our results demonstrate that sepsis induced an upregulation of TLR4 and MCP1 immunoreactivity in neuroanatomical areas of the rostral NTS, an effect that was further exacerbated by morphine. The observed parallel between the morphine-induced enhancement of TLR4/MCP1 expression in the NTS and the concurrent suppression of BRS_PE_ suggests a potential causal link between the two effects. Moreover, the ability of naloxone and pharmacological MAPK/ROCK inhibitors to prevent the morphine-induced upregulation of TLR4 and MCP1 points to the involvement of these molecular pathways in mediating the µ-opioid receptor-mediated baroreceptor dysfunction induced by morphine during sepsis. It should be noted that the NTS was specifically targeted due to its major role in receiving and central processing peripheral inflammatory (Waki et al., 2008) and arterial baroreceptor signals (Accorsi-Mendonça and Machado, 2013). Importantly, both TLR4 and MCP1 are expressed in the NTS, and their upregulation has been shown to contribute to cardiovascular dysfunction associated with endotoxemia (Paxinos and Watson, 1986; Nakano et al., 2015; Abuiessa et al., 2020; Sallam et al., 2021b). Furthermore, the identification of opioid receptors and neurons within the NTS (Reeves et al., 2022) supports the potential neuromodulatory role of opioid signaling in modulating the baroreflex-inflammatory axis. It is noteworthy that we focused on the rostral NTS in the present study because our previous findings reported that this region plays a central role in integrating cardiovascular and autonomic responses during septic inflammation (Paxinos and Watson, 1986; Nakano et al., 2015; Abuiessa et al., 2020; Sallam et al., 2021b). Given that both the caudal and rostral NTS are involved in the central regulation of cardiac and neuroinflammatory processes (Phillis et al., 1997; Pereyra et al., 2025), The role of caudal NTS neurons in the CLP/morphine interaction warrants further study to determine potential region-specific differences in inflammatory signaling.

Our finding that morphine increased TLR4 and MCP1 expression in the NTS appears contradictory to the widely recognized immunosuppressive effects of opioids, including morphine (Bettinger and Friedman, 2024). Nonetheless, it is notable that the immunomodulatory effects of morphine are multifaceted and vary depending on the physiological context. In peripheral tissues, morphine is generally immunosuppressive, reducing natural killer (NK) cell activity, inhibiting T and B lymphocyte proliferation, and decreasing cytokine production (Sacerdote, 2006; Liang et al., 2016). In contrast, morphine enhances central neuroinflammation through the binding to TLR4 on microglia, leading to the release of proinflammatory cytokines, and disrupting glutamate transporters (Wang et al., 2012; Eidson et al., 2017).

It is important to comment on two potential limitations of the current study. First, the arterial baroreflex activity was not assessed before individual drug treatments. The absence of baseline baroreflex measurements prevented each rat from serving as its own control, which could have helped minimize the influence of inter-animal variability stemming from differences in basal baroreflex activity. This approach was not feasible in our study because generating two full dose-response curves for phenylephrine and sodium nitroprusside (i.e., before and after individual drug treatments) would necessitate i.v. administration of relatively large volumes of the drug solutions, along with additional flushing between doses. This may potentially cause acute volume overload, a condition that may act in itself to alter autonomic and arterial baroreflex activities (Wong and Johns, 1999; Raimundo Fernades et al., 2020) and perhaps confound data interpretation. Remarkably, spontaneous baroreceptor sensitivity measurement is an alternative to the traditional Oxford vasoactive method. The spontaneous approach exploits natural beat-to-beat fluctuations in systolic blood pressure and the corresponding reflex changes in heart rate or RR intervals observed at rest (Pinna et al., 2015). This method avoids pharmacological interventions, provides insight into real-time physiological baroreflex function, and allows for long-term baroreflex monitoring. However, unlike the vasoactive method, which assesses the full range of baroreflex function, the spontaneous method is limited by its dependence on adequate intrinsic BP variability and its focus on responses around the resting pressure range. Such narrower dynamic range may lead to underestimation of baroreflex sensitivity (Laude et al., 2008; Milic et al., 2009).

The second limitation of the present study concerns the possibility that the analgesia induced by morphine upon i.v. administration may have contributed to its interaction with arterial baroreflex activity. The postoperative pain induced by invasive surgical procedures such as femoral catheterization and laparotomy are believed to be coupled with elevated sympathetic drive, which typically persists for 24–48 h postoperatively and can interfere with cardiovascular function and reflexes (Roughan and Flecknell, 2001; Leach et al., 2011; Zubrzycki et al., 2018). Considering that morphine administration relieves the nociceptive pain and reduces the sympathetic drive (Pertovaara and Wei, 2001; Baby et al., 2021), the possibility cannot be overlooked that the attenuation of baroreflex activity observed in our study could reflect both direct pharmacological effects of morphine on cardiovascular autonomic regulation, and indirect effects mediated via analgesia and reduced nociceptive input. Further studies are warranted to unravel these contributions.

Together, we demonstrate that acute morphine exposure selectively amplifies the sepsis-induced impairment of baroreceptor-mediated bradycardic, but not tachycardic, responses. Pharmacological interventions and protein expression analyses implicate brainstem inflammatory and chemotactic pathways within the neuroanatomical area of the NTS, along with downstream MAPK and ROCK signaling, in mediating the baroreflex effects of morphine. Accordingly, the interruption of these signaling pathways may offer a therapeutic strategy to maintain cardiovascular reflex integrity in patients receiving opioids, particularly in pathological states where baroreflex function is already impaired like sepsis.

## Data Availability

The original contributions presented in the study are included in the article/Supplementary Material, further inquiries can be directed to the corresponding author.
